# A Case Report of Neuropathic Pain and Unilateral Lower Extremity Weakness Following a Cardiac Arrest

**DOI:** 10.7759/cureus.50240

**Published:** 2023-12-09

**Authors:** Kashif N Malik, Justin Chan, Brian Vu

**Affiliations:** 1 Physical Medicine and Rehabilitation, Casa Colina Hospital, Pomona, USA; 2 Pain Management, Western University of Health Sciences, Pomona, USA

**Keywords:** percutaneous coronary intervention, iatrogenic femoral neuropathy, neuropathic pain, physical medicine and rehabilitation, pain management, femoral nerve palsy

## Abstract

Femoral nerve palsy is a rare, but significant complication following percutaneous coronary intervention (PCI) for conditions such as myocardial infarction. We present a case of a 61-year-old male patient who presented for cardiac rehabilitation following an emergent PCI procedure for cardiac arrest secondary to ST-elevation myocardial infarction. He later developed right lower extremity weakness and severe neuropathic pain on arrival to the acute rehabilitation unit. After physical examination and electrodiagnostic studies, he was determined to have a right femoral nerve neuropathy. This case report highlights the clinical course, physical examination/electrodiagnostic findings, and subsequent pain management of femoral nerve palsies.

## Introduction

Femoral nerve palsy is a condition that results from injury or damage to the femoral nerve. The femoral nerve originates from the dorsal-ventral rami of the second, third, and fourth lumbar nerves. It then provides motor and sensory innervation to the anterior thigh muscles and part of the lower leg [1].

The condition presents with weakness, numbness, and occasional pain in the distribution of the femoral nerve. This typically affects the anterior aspect of the thigh and can sometimes extend to the medial aspect of the lower calf. Initial symptoms include difficulty in hip flexion and knee extension and altered sensation over the anterior thigh. These deficits can lead to gait dysfunction and reduce an individual’s function by limiting their ability to ambulate and transfer.

Approximately 600,000 to 900,000 percutaneous interventions are performed in the United States annually [2]. An analysis of approximately 15,894,201 patients who underwent percutaneous coronary intervention (PCI) from 2002 to 2010 revealed that 597 patients suffered a femoral nerve injury as a result of the procedure [2]. This resulted in approximately 3.8 injuries per 100,000 procedures. Patients with congestive heart failure or coagulopathy had a significantly higher rate of injury compared to those who did not.

Percutaneous interventions are minimally invasive medical procedures that involve the use of a small incision or needle punctures to access the femoral artery in order to treat medical conditions such as coronary and peripheral artery disease [2]. In the United States, around 900,000 of these treatments are utilized per year; however, one potential complication associated with this treatment is the incidence of femoral palsy [2]. Femoral palsy is a rare but significant complication characterized by weakness or paralysis of the leg due to injury of the femoral nerve during the procedure leading to an increased likelihood of a moderate-to-severe disability at the patient’s discharge.

Femoral nerve palsy can result from various causes, including surgical trauma, compression, and entrapment, inflammatory or infectious conditions [3]. During surgical procedures such as coronary artery bypass grafting (CABG), particularly with the use of femoral artery cannulation, the femoral nerve may be inadvertently injured or compressed. Prolonged pressure on the femoral nerve due to positioning during surgery, prolonged immobility, or compression from nearby structures can also lead to nerve injury. Femoral nerve injury can either result from direct injury to the nerve via cannulation or through the development of a hematoma or pseudoaneurysm [2]. Inflammatory processes, infections, or autoimmune disorders can directly affect the nerve or its surrounding structures and can cause nerve injury.

The diagnosis of femoral nerve palsy involves a thorough clinical assessment, including history, physical examination, and often electrodiagnostic tests such as electromyography (EMG) and nerve conduction studies (NCS). Imaging modalities such as magnetic resonance imaging (MRI) or computed tomography (CT) may be used to visualize the nerve and rule out other structural issues in rare cases.

## Case presentation

The patient is a 61-year-old male who presented to acute inpatient rehabilitation for deconditioning and deficits in mobility and activities of daily living (ADLs) related to a recent cardiac arrest. His past medical history included polysubstance abuse, coronary artery disease (CAD), and hypertension. The patient was found unresponsive in his house. Emergency Medical Services (EMS) arrived at the scene, and the patient was unresponsive with agonal respirations. He was found to be in ventricular fibrillation, and he did receive cardiopulmonary resuscitation (CPR) as well as electric cardioversion, however, he remained in ventricular fibrillation.

He was intubated en route to the hospital. He did achieve the return of spontaneous circulation, however, he did subsequently lose his pulse approximately five times during his emergency room (ER) work-up and required subsequent resuscitation.

He was emergently taken to the cardiac cath lab for an ST-elevation myocardial infarction and underwent PCI with access through his right groin. He also continued to require ventilator support after the procedure. He was found to have acute renal failure, and nephrology was consulted who placed the patient on hemodialysis.

He did have another two episodes of asystole and subsequent return of spontaneous circulation (ROSC) during his hospitalization. He was maintained on multiple pressor agents for blood pressure support, which were later weaned during his stay. An echocardiogram showed left ventricular ejection fraction (LVEF) of 40% to 45% with grade 1 diastolic dysfunction.

His hospital course was then complicated by *Pseudomonas* pneumonia and was started on IV antibiotics.

He was eventually decannulated, transitioned to high-flow oxygen, and then weaned to supplemental oxygen as needed. He was evaluated by physical and occupational therapy and was found to have extensive deficits in mobility and ADLs. He was felt to be a good candidate for acute inpatient rehabilitation given his deficits.

He then presented to acute rehabilitation and was noted to have right lower extremity weakness and severe neuropathic pain on admission.

His initial physical exam is documented.

Right lower extremity

Hip flexion 2/5, hip extension 5/5, knee flexion 5/5, knee extension 3/5, ankle dorsiflexion 5/5, ankle plantarflexion 5/5

Left lower extremity

Hip flexion 5/5, hip extension 5/5, knee flexion 5/5, knee extension 5/5, ankle dorsiflexion 5/5, ankle plantarflexion 5/5

Sensory

Impaired to light touch and pinprick over the right anterior thigh, otherwise intact in the bilateral upper and lower extremities.

Deep tendon reflexes (DTRs)

Right biceps 2+, triceps 2+, BR 2+, KJ 1+, AJ 2+, toes downgoing

Left biceps 2+, triceps 2+, BR 2+, KJ 2+, AJ 2+, toes downgoing

**Figure 1:**
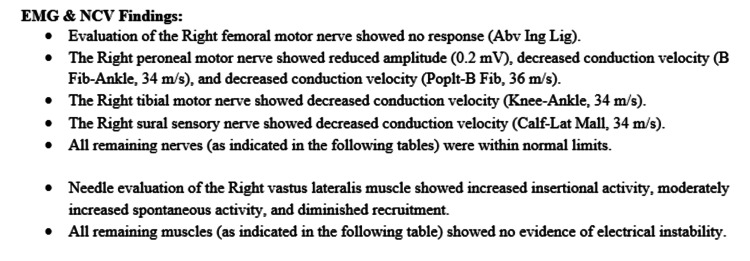
Electromyography (EMG) and Nerve Conduction Study (NCS) Findings

**Figure 2:**
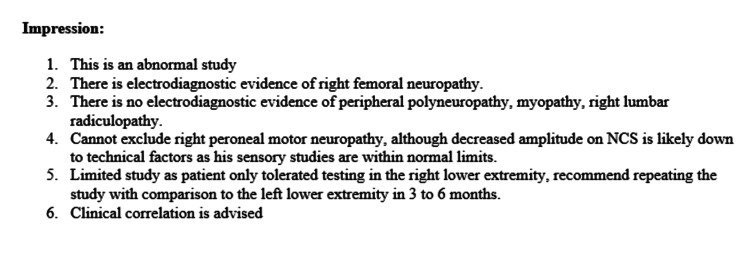
Electromyography (EMG) and Nerve Conduction Study (NCS) Impressions

During his stay, an EMG and NCS, as seen in Table 1, were performed to determine the etiology of his right lower extremity weakness and dysesthesias. The impressions of the test are listed in Figure 1 and Figure 2, respectively. The NCS and EMG showed findings that were consistent with femoral nerve neuropathy, including a lack of femoral motor nerve response on motor NCS, and increased insertional activity with fibrillations and positive sharp waves on EMG testing in the vastus lateralis muscle, which receives its innervation from the femoral nerve.

**Table 1:** Nerve Conduction Study and Electromyography (EMG) Results

Site	No Response	Peak (ms)	Norm Peak (ms)	P-T Amp (µV)	Normal P-T Amplitude	Site1	Site2	Delta-P (ms)	Distance (cm)	Velocity (m/s)	Normal Velocity (m/s)
Right Superficial Peroneal Anti Sensory (Anterior Lateral Malleolus)											
14 cm		3.8	<4.0	36.8	>5.0	14 cm	Ant Lat Mall	3.8	14.0	37	
Right Sural Anti Sensory (Lateral Malleolus)											
Calf		No Response									

Site	No Response	Onset (ms)	Normal Onset (ms)	O-P Amplitude (mV)	Norm O-P Amplitude	Site1	Site2	Delta-0 (ms)	Distance (cm)	Velocity (m/s)	Normal Velocity (m/s)
Right Femoral Motor (Vastus Medialis)											
Above Inguinal Ligament	No Response										
Below Inguinal Ligament		13.8		0.0							
Right Peroneal Motor (Extensor Digitorum Brevis)											
Ankle		6.0	<6.5	0.2	>1.3	B Fib	Ankle	10.1	34.0	34	>38
Below Fibula		16.1		0.2		Poplt	B Fib	2.8	10.0	36	>40
Popliteal Fossa		18.9		0.3							
Right Tibial Motor (Abductor Hallucis Brevis)											
Ankle		6.1	<6.1	6.5	>4.4	Knee	Ankle	12.7	43.0	34	>39
Knee		18.8		5.0							

EMG Side	Muscle	Nerve	Root	Insertional Activity	Fibrillations	Positive Sharp Waves	Amplitude	Duration	Polys	Recruitment	Effort	Comment
Right	Vastus Lateralis	Femoral	L2-4	Increased	1+	2+	Normal	Normal	0	Reduced	Normal	
Right	Biceps Femoris	Sciatic	L5-S1	Normal	Normal	Normal	Normal	Normal	0	Normal	Normal	
Right	Anterior Tibialis	Deep Peroneal	L4-5	Normal	Normal	Normal	Normal	Normal	0	Normal	Normal	
Right	Medial Gastrocnemius	Tibial	S1-2	Normal	Normal	Normal	Normal	Normal	0	Normal	Normal	

The patient continued with a comprehensive rehabilitation program that focused on strengthening and mobility for his right lower extremity.

Neuropathic pain in the patient’s right lower extremity was a barrier for the patient during his stay. He initially was started on gabapentin 100mg TID during his hospital stay, which did provide partial relief for the patient’s symptoms. Caution was exercised with the dosage to avoid side effects given that the patient was on multiple other sedating medications and had a previous renal injury.

After discharge the patient improved his strength to 4-/5 for right hip flexion and 3/5 in right knee extension. At his one-month follow-up, the patient continued to endorse burning and dysesthesias in his right anterior thigh which was worse at night. His gabapentin was then titrated to 300mg TID, with instructions to take 300mg at night initially for 3-5 days then titrate to the TID dosage. Duloxetine 30mg daily was also added as the patient was endorsing some depressive symptoms and continued nerve pain.

At his second follow-up visit two months after discharge, the patient reported some reduction in his pain, however, was continued to endorse similar symptoms. His gabapentin was then increased to 600mg nightly with 300mg twice a day. Tramadol 50mg q8h as needed was added for breakthrough pain, and his duloxetine was continued. The patient continued to progress in terms of his lower extremity strength.

On his initial inpatient physical therapy evaluation, the patient’s 5xSTS (sit to stand x 5 reps) time was 36.65 seconds, and required bilateral upper extremity support and bracing in his lower extremities as well. Three months later in outpatient physical therapy, his 5xSTS time decreased significantly to 10.59 seconds with no support required.

Additionally, his 10M walk test improved from 18 seconds (gait velocity 0.33 m/s) with a front wheel walker on admission to 6.49 seconds (0.92 m/s) without an assistive device three months following his discharge.

## Discussion

Femoral nerve palsy is an important, yet underrecognized complication following PCI and other procedures where femoral artery cannulation occurs. The femoral nerve is a separate and distinct nerve originating from the lumbar spine but travels down the front of the thigh [4].

Femoral nerve palsy refers to the compression of the femoral nerve during the PCI procedure leading to compression of the femoral nerve due to a catheter or other equipment pressing against it, resulting in pain, weakness, and sensory disturbances in the front of the thigh. Many patients may also be dealing with pre-existing conditions such as herniated discs or spinal stenosis leading to increased risk factors [5]. Lumbar radiculopathy refers to compression or irritation of spinal nerve roots leading to pain, numbness, and weakness in the lower back and down one leg [6]. Both have distinct conditions with different underlying causes, therefore, it is essential to accurately diagnose the underlying cause to determine the appropriate treatment for these conditions.

Treatment involves physical therapy to strengthen the affected muscles, improve mobility, and aid in nerve recovery. In severe cases or when conservative measures fail, surgical interventions such as nerve decompression or repair are considered [7]. The individual's prognosis varies based on the extent of nerve damage, their initial clinical condition, and the effectiveness of the chosen treatment approach.

A comprehensive evaluation, including history and physical examination, is required to assess the extent and severity of femoral nerve palsy, including motor and sensory deficits, muscle strength, range of motion, and functional limitations. Scales can be used to assess patients' level of independence such as the Functional Independence Measure scores.

Physical therapy management includes improving muscle strength and function in the affected lower limb, enhancing mobility, gait, and overall functional abilities, and alleviating pain and discomfort associated with femoral nerve palsy [8]. Exercises include active and passive range of motion exercises such as resisted knee extensions for the affected lower limb to maintain flexibility, improve muscle coordination, and prevent contractures. Other exercises include resistance training with bands to target hip flexors and quadricep muscles.

Therapists employ strengthening exercises targeting the quadriceps, hip flexors, and lower limb muscles to improve muscle tone and strength and incorporate resistance training, including weight-bearing exercises and resistance bands, to progressively increase muscle strength. Assistive devices like canes or walkers are often used as needed to aid in balance and stability during gait training. Modalities such as electrical stimulation to the affected muscles promote muscle contraction, improve circulation, and expedite the healing process.

Pain management is a critical component in the management of femoral nerve palsy. Neuropathic pain can be disabling to patients and limit their functioning and recovery. Typical management includes physical therapy, non-steroidal anti-inflammatory drugs (NSAIDs), and gabapentinoids [9]. For refractory cases, adjunct agents such as duloxetine and tricyclic antidepressants can be added. Rarely, patients will have persistent pain and debility that will require the addition of stronger medications such as tramadol and opioids [9]. There have been case reports of patients with excruciating femoral neuropathy requiring peripheral nerve stimulator placement under ultrasound [9].

## Conclusions

In conclusion, this case report of severe neuropathic pain from femoral nerve palsy following a PCI highlights the importance of pain management and rehabilitation in the management of this rare and often under-recognized complication. The rehabilitation process for femoral nerve palsy requires a multidisciplinary approach, involving physical therapists, occupational therapists, and physicians, to create a patient-specific rehabilitation program. Emphasis should be placed on muscle strengthening, range of motion exercises, gait training, and neuromuscular re-education to target the affected extremity and improve motor function. Pain management is a crucial aspect of this condition and involves close titration of medications and the development of a rehabilitation protocol to maximize the patient's function and reduce the symptom burden.

Furthermore, continued research and collaboration within the medical community are essential to develop standardized guidelines and evidence-based rehabilitation protocols specific to pain management and rehabilitation for femoral nerve palsy following PCI. This will aid in optimizing rehabilitation outcomes, advancing patient care, and mitigating the functional impact of this uncommon but significant complication associated with PCIs.
